# Anti-tumor effect of ribavirin in combination with interferon-α on renal cell carcinoma cell lines *in vitro*

**DOI:** 10.1186/1475-2867-14-63

**Published:** 2014-09-03

**Authors:** Lichen Teng, Dexin Ding, Yongsheng Chen, Hongshuang Dai, Guobin Liu, Zhongjie Qiao, Ruihua An

**Affiliations:** Department of Urology, The Affiliated Tumor Hospital, Harbin Medical University, No. 150 Haping Road, Harbin city, Heilongjiang Province 150081 China; Department of Urology, The First Affiliated Hospital, Harbin Medical University, No. 31 Youzheng Street, Harbin city, Heilongjiang Province 150080 China

**Keywords:** Ribavirin, RCC, Proliferation, Apoptosis, Migration

## Abstract

**Background:**

Ribavirin is an anti-viral drug; however, recent data suggest that it may also be effective in cancer therapy. This study investigated the effect of ribavirin alone or in combination with IFN-α on biological processes: proliferation, apoptosis, and migration of murine (Renca) and human renal carcinoma (RCC) cells (786–0) *in vitro*.

**Methods:**

Renca and 786–0 cells were treated with IFN-α, ribavirin, or a combination of IFN-α and ribavirin at varying concentrations. Cell proliferation was evaluated using CCK-8 assay. Induction of apoptosis and distribution of cell cycle were determined by flow cytometry. The migratory capacity of cells was quantified using a transwell migration assay. The toxic effect of these drugs was examined using MTT assay in HEK-293 cells. ELISA was used to measure IL-10 and TGF-β content in the culture supernatants.

**Results:**

Our results showed that both ribavirin alone and in combination with IFN-α could significantly inhibit the cell proliferation and arrest the cell cycle progress at the G2/M phase. These treatments also inhibited cell migration and IL-10 production, in a concentration-dependent manner, in 786–0 and Renca cells. Moreover, they significantly induced apoptosis of RCC cells and increased TGF-β production in concentration-dependent manner. No significant toxic effect was observed in HEK-293 cells. We also found that the effect of combined treatment was more pronounced than that of ribavirin or IFN-α alone. However, the combined effect of the two drugs was not synergistic.

**Conclusion:**

Our findings suggest that ribavirin can negatively affect biological processes of RCC cells. This agent might become a new candidate for the treatment of RCC in the clinical setting.

## Background

Renal cell carcinoma (RCC) is one of the cancers most resistant to currently available chemotherapy, radiotherapy, and immunotherapy. Although nephrectomy and nephron-sparing surgery (NSS) are still the mainstream therapies in RCC, satisfactory outcomes are only achieved in patients with localized RCC. A Canadian study has demonstrated that at the time of first diagnosis, 25% of patients with RCC are diagnosed with locally advanced RCC with lymph node or local organ involvement, and 30% would present with metastasis [1]. Two decades ago, cytokine therapy, such as IL-2 or interferon alone or administered together, were typically used in the initial RCC therapy. The patients with the advanced or metastatic RCC would need a combination of surgery and immunotherapy. Unfortunately, only a small proportion of such patients show significant and durable response to immunotherapy. Since the advent of targeted therapy, tyrosine kinase inhibitors, such as sorafenib and sunitinib, have attracted worldwide attention, bringing new hope to RCC patients. However, target therapy rarely gives a complete response in patients with localized or metastatic RCC. In addition, because of the high cost and severe side effects of such interventions, these drugs have not been widely used in China.

Ribavirin (1-β-D-ribofuranosyl-1,2,4-triazole-3-carboxamide) is a well-known anti-virus drug, widely used in the treatment of various viral infections, especially hepatitis-C infections [2, 3]. This drug can markedly improve outcome for patients with hepatitis C; nevertheless, its precise mode of action has remained elusive. Some researchers have focused on potential immunomodulatory properties of ribavirin. A recently published study has demonstrated that ribavirin can inhibit the function of HCV-specific regulatory T cells and reverse the suppression of T effector cells [4]. The study suggests that ribavirin might exert a similar immunomodulatory effect in anti-cancer therapies. Some studies have shown that ribavirin can inhibit the activity of oncogenic eukaryotic translation-initiation factor eIF4E [5–8]. All these data have triggered our renewed interest in the potential effect of ribavirin on cancer cells.

Interferon-α (IFN-α) has shown anti-tumor activity in a variety of solid tumors and has been approved for RCC treatment. However, the available evidence demonstrates that IFN-α only produces modest benefits in unselected patients; randomized clinical trials show only a small survival benefit [9, 10]. This limited efficacy of IFN-α treatment has narrowed its application as a single agent in patients with RCC. Some investigators have even concluded that subcutaneous IFN-α should not be recommended for patients with metastatic RCC. However, it is worth considering combining IFN-α with other agents. This approach might enhance the anti-tumor effect of IFN-α in RCC. For example, combined ribavirin and IFN-α treatment might have a synergistic effect in the management of RCC. This study investigated the inhibitory effect of ribavirin and IFN-α on human and mouse RCC cell lines *in vitro*.

## Results

### The effect of IFN-α, ribavirin, or a combination of IFN-α and ribavirin on the proliferation of renca and 786–0 cells

**Figure 1:**
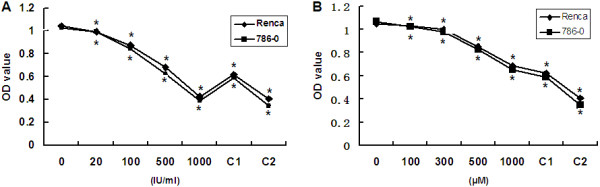
**The effect of IFN-α and ribavirin alone and combined IFN-α and ribavirin treatment on the proliferation of the murine and human RCC cells. (A)** Suppression of proliferation induced by IFN-α alone or in combination with ribavirin. **(B)** Suppression of proliferation induced by ribavirin alone or in combination with IFN-α. C1: combination of 500 IU/ml of IFN-α and 500 μM ribavirin. C2: 1000 IU/ml of IFN-α and 1000 μM ribavirin. The suppression was significant in the range of 20–1000 IU/ml of IFN-α or 100–1000 μM ribavirin. (*indicates a significant difference in comparison with controls).

Renca and 786–0 cells were treated with IFN-α, ribavirin, or a combination of IFN-α and ribavirin for 72 h. We observed significant decrease in OD values with increasing concentration of IFN-α (0, 20, 100, 500, 1000 IU/ml) or ribavirin (0, 100, 300, 500, 1000 μM) in both cell lines (p < 0.05). These data demonstrate that the cell proliferation can be suppressed by IFN-α or ribavirin in a concentration-dependent manner. To investigate whether combined treatment can have a synergistic effect on the proliferation of these cells, the cell cultures were incubated with IFN-α and ribavirin at different concentrations (IFN-α at 500 or 1000 IU/ml and ribavirin at 500 or 1000 μM). We observed that, in comparison with ribavirin-only treatment, combined treatment significantly inhibited the proliferation of Renca and 786–0 cells in a concentration-dependent manner (p < 0.05). However, for both cell lines, the combined treatment with 500 IU/ml of IFN-α and 500 μM ribavirin produced OD values higher than after incubation with 1000 IU/ml of IFN-α. In addition, the OD values reached 0.404 and 0.345 at the concentrations of 1000 IU/ml of IFN-α and 1000 μM ribavirin in Renca and 786–0 cells, respectively. The results are shown in Figure 1A and B. The results demonstrate that the combination of IFN-α and ribavirin has stronger inhibitory effect on RCC cell growth than incubation with ribavirin alone. However, the confidence interval (CI) values showed an additive inhibitory effect when RCC cell lines were treated with IFN-α and ribavirin.

### The effect of IFN-α alone, ribavirin alone, or a combination of IFN-α and ribavirin on apoptosis of human and murine RCC cells

**Figure 2:**
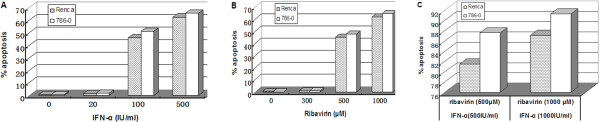
**Apoptosis in human and murine RCC cells after treatment with IFN-α alone, ribavirin alone, or combination of IFN-α and ribavirin. (A)** Apoptosis induced by IFN-α alone. **(B)** Apoptosis induced by ribavirin alone. **(C)** Apoptosis induced by combination of IFN-α and ribavirin.

Our results showed that IFN-α or ribavirin alone can induce apoptosis of RCC cells. However, the effect at lower concentrations of those agents was slight; only 1–2% apoptosis was observed in the cells treated with 20 IU/ml IFN-α or 300 μM ribavirin. With higher concentrations of IFN-α or ribavirin, we observed significantly increasing apoptosis rate, to a maximum of 60–70% at 500 IU/ml of IFN-α or 1000 μM ribavirin (Figure 2A and B). Thus, the effect of these agents on apoptosis was concentration-dependent. Interestingly, higher percentages of apoptotic cells (80–92%) were observed in RCC cells treated with a combination of IFN-α and ribavirin at high concentration levels (Figure 2C). CompuSyn analysis showed that combined treatment with IFN-α and ribavirin had an additive effect on apoptosis in RCC cells.

### The effect of ribavirin and IFN-α on 786–0 cell cycle

**Figure 3:**
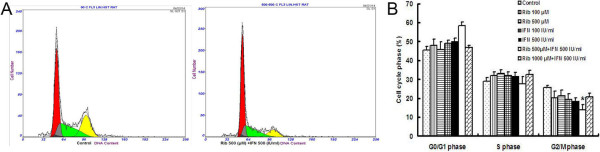
**Combined treatment with ribavirin and IFN-α affected the RCC cell cycle. (A)** Combination of 500 IU/ml of IFN-α and 500 μM ribavirin blocked the G2/M phase cell cycle transition of 786–0 cells, in comparison with untreated control 786–0 cells. The percentage of 786–0 cells in the G0/G1 phase increased significantly to 58.32% but the percentage of cells in the G2/M phase decreased to 13.95%. **(B)** Quantitative analysis of the cell cycle distribution of 786–0 cells treated with ribavirin alone, IFN-α alone, or a combination of ribavirin and IFN-α (* < 0.05 versus control group).

Apart from the inhibition of proliferation and induction of apoptosis in RCC cells, we also investigated the effect of ribavirin and IFN-α on 786–0 cell cycle. Our results, shown in Figure 3A and B, demonstrate that the percentages of 786–0 cells in the G0/G1 and S phase were similar in treated and untreated samples. With increasing concentration, however, the percentage of 786–0 cells in the G2/M phase decreased significantly, from 25.52% to 13.95%. The percentage of cells in the G2/M phase was lowest (13.95%) after treatment with 500 μM of ribavirin and 500 IU/ml of IFN-α. These results suggest that ribavirin and IFN-α induce the cell cycle arrest at the G2/M phase. CompuSyn analysis revealed an additive effect of the two agents on the cell cycle arrest.

### Cell cytotoxicity of ribavirin and IFN-α in the human embryonic kidney cell line (HEK-293)

**Figure 4:**
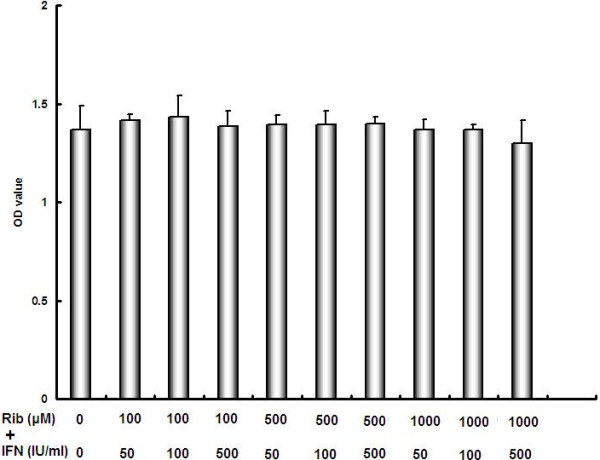
**Combination of ribavirin and IFN-α does not cause significant cell toxicity in HEK-293 cells, in comparison with untreated cells.**

To examine cytotoxicity of ribavirin and IFN-α in a normal cell line, the HEK-293 cells were co-treated with these two agents. The cytotoxicity was then examined using the MTT assay. After 72-h incubation, the proliferation of HEK-293 cells was not significantly affected by combined treatment with different concentrations of ribavirin and IFN-α. The results, shown in the Figure 4, demonstrated that this treatment did not have significant cytotoxic effect in the normal kidney cell line.

### The effect of IFN-α alone, ribavirin alone, or a combination of IFN-α and ribavirin on RCC cell migration

**Figure 5:**
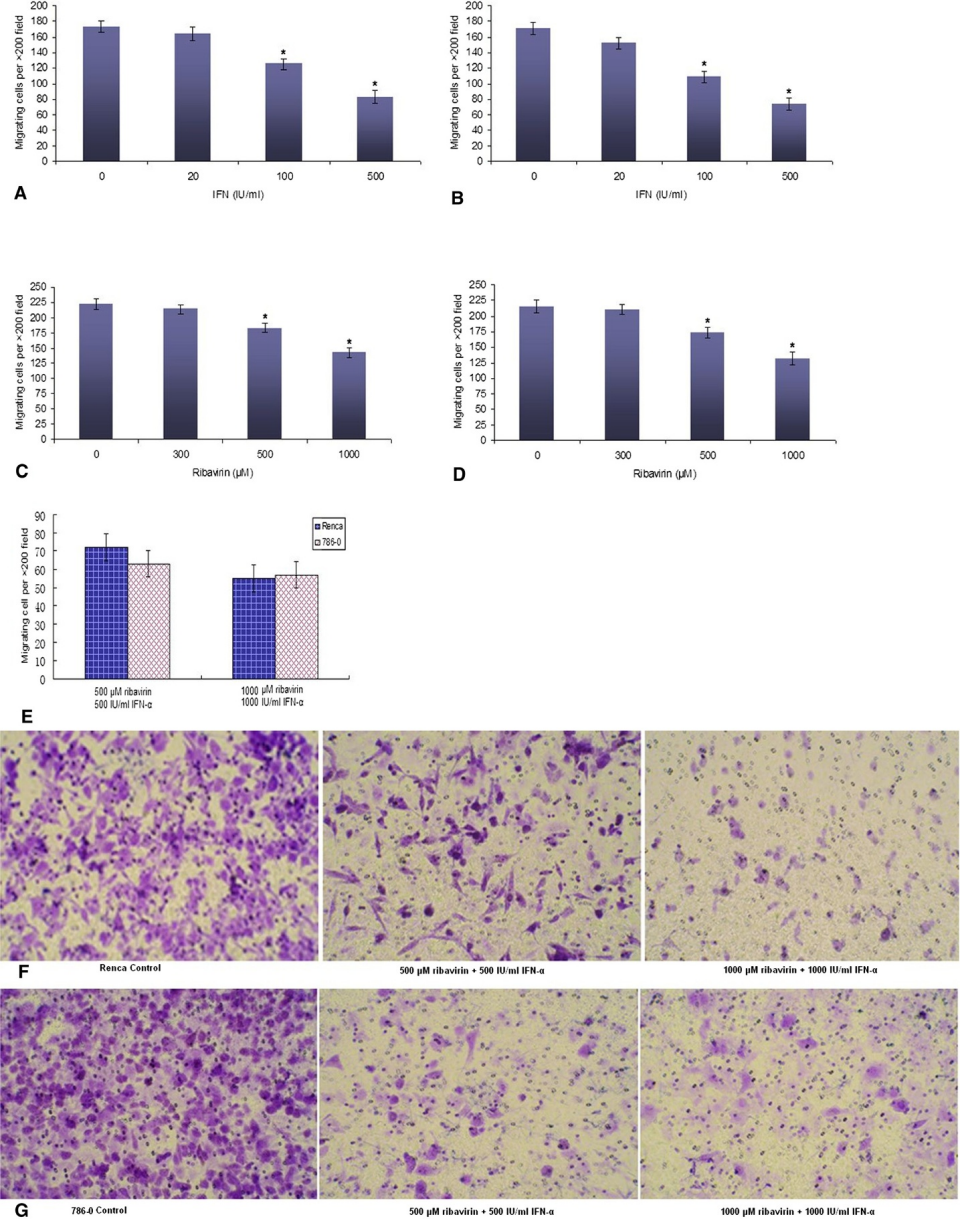
**Ribavirin alone, IFN-α alone, or a combination of IFN-α and ribavirin inhibit migration of Renca or 786–0 cells. (A)** The effect of IFN-α on migration of Renca cells **(A)** and 786–0 cells **(B)**; The effect of ribavirin on migration of Renca cells **(C)** and 786–0 cells **(D)**; **(E)** The effect of combined IFN-α and ribavirin treatment on migration of Renca and 786–0 cells (*indicates a significant difference in comparison with controls). **(F, G)** Representative images of crystal violet-stained control and treated Renca and 786–0 cells (combined treatment with IFN-α and ribavirin at different concentrations).

The transwell assay showed a significant decrease in cell migration for the cells incubated with IFN-α in comparison with untreated cells, for both Renca and 786–0 cell lines. This effect was concentration-dependent for both cell lines (p < 0.05) (Figure 5A and B). The treatment with ribavirin significantly inhibited cell migration in both cell lines in a similar manner (p < 0.05) (Figure 5C and D), but the effect of ribavirin on cell migration was weaker than that of IFN-α. Surprisingly, after combined IFN-α and ribavirin treatment, fewer cells (50–70) underwent migration (p < 0.05) (Figure 5E-G). The results showed that both agents exerted a potent effect on cell migration. However, in comparison with the results for the combined treatment with 500 μM of ribavirin and 500 IU/ml of IFN-α, increasing IFN-α concentration caused no further decrease in the number of migrating cells (in both cell lines, p > 0.05).

### The effect of IFN-α alone, ribavirin alone, or combined treatment on IL-10 and TGF-β secretion in supernatant of RCC cells

**Figure 6:**
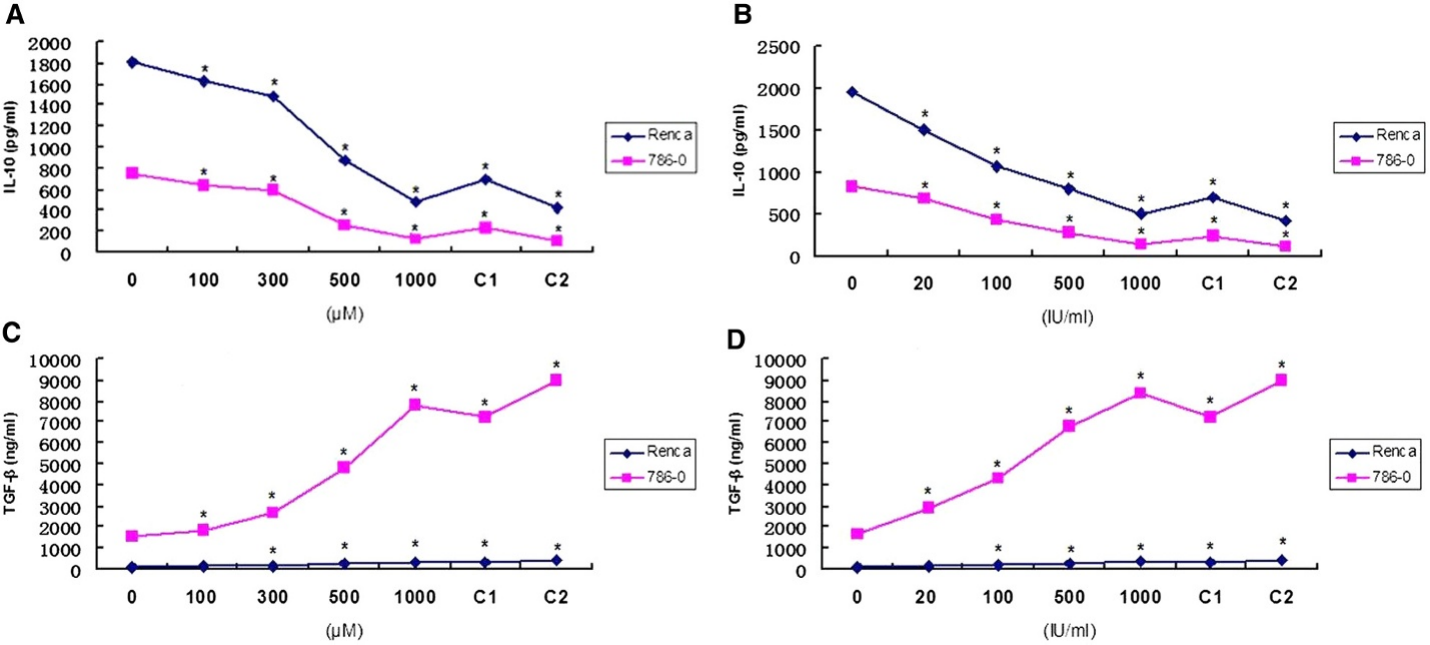
**IFN-α alone, ribavirin alone, or a combination of IFN-α and ribavirin inhibit IL-10 production, but increase TGF-β secretion in Renca and 786–0 cells. (A)** IL-10 levels after treatment with ribavirin (100–1000 μM) or a combination of IFN-α and ribavirin. **(B)** Levels of IL-10 after treatment with IFN-α (20–1000 IU/ml) or a combination of IFN-α and ribavirin. **(C)** Levels of TGF-β after treatment with ribavirin (100–1000 μM) or a combination of IFN-α and ribavirin. **(D)** Levels of TGF-β after treatment with IFN-α (20–1000 IU/ml) or a combination of IFN-α and ribavirin. C1: combination of 500 IU/ml of IFN-α and 500 μM ribavirin. C2: 1000 IU/ml of IFN-α and 1000 μM ribavirin. The suppression was significant within the range of 20–1000 IU/ml of IFN-α or in the range of 100–1000 μM ribavirin (*indicates a significant difference in comparison with controls).

IL-10 concentrations in supernatants of Renca and 786–0 cells treated with ribavirin at different concentrations were significantly lower (p < 0.05) than in the supernatants from untreated cultures. These results demonstrate that ribavirin can inhibit IL-10 secretion in RCC cells in a concentration-dependent manner (Figure 6A). Similarly, IFN-α decreases IL-10 secretion in both treated RCC cell lines. However, the treatment with a combination of IFN-α and ribavirin at higher concentration levels did not inhibit IL-10 secretion more than single-agent treatments (Figure 6A and B). In contrast, there was a trend towards increased TGF-β production. When TGF-β levels were measured with ELISA, we observed that both IFN-α and ribavirin on their own significantly increased TGF-β production in a concentration-dependent manner (in both Renca and 786–0 cells, p < 0.05). The combined treatment did not significantly increase TGF-β secretion. We also found that the increase in TGF-β production caused by IFN-α or ribavirin was more substantial in 786–0 cells than that in Renca cells (Figure 6C and D).

## Discussion

The aim of this study was to evaluate the direct effect of ribavirin on RCC cells and the possibility that this agent might become a new candidate for RCC therapy. Ribavirin is a classic anti-viral agent and has been used for the treatment of various viral diseases for a long time. Previous studies have demonstrated that IFN-α alone or in combination with other agents can directly inhibit the growth of cancer cells. However, up to now, ribavirin has not been used to treat RCC [11, 12]. Our *in vitro* experiments revealed that ribavirin could directly inhibit proliferation and migration of murine and human RCC cells, enhance their apoptosis, and arrest cell cycle progression at the G2/M phase. The effect of ribavirin on RCC cells was concentration-dependent. Our results also showed that ribavirin inhibited IL-10 production and increased TGF-β secretion. More importantly, combined treatment with IFN-α and ribavirin had an additive effect on the proliferation, migration, and induction of apoptosis. In this study, we did not observe significant toxic effects on normal cells. Thus, our results suggest that ribavirin might be a novel agent for the treatment of RCC.

The direct anti-tumor effect of ribavirin cells certainly deserves further investigation. Increasing ribavirin concentration significantly increased the efficacy of its anti-cancer properties *in vitro*. We found that ribavirin (at high concentration levels of 500 and 1000 μM) strongly inhibited proliferation, induced apoptosis, and inhibited cell migration. The level of transporter expression in RCC cells and intracellular levels of ribavirin and its metabolites are likely to be interrelated. It has been suggested that ribavirin is taken up by nitrobenzylthioinosine-sensitive (es)-nucleoside transporters and that the concentrative nucleoside transporters and sodium co-transport could be important at low ribavirin concentrations [13, 14]. High intracellular levels of ribavirin and its metabolites also depend on the levels of ribavirin in the extracellular media [15, 16]. Detailed studies of ribavirin metabolism and its transport processes in the RCC cells, which might modulate intracellular levels of ribavirin, will be important for understanding the exact mechanisms of its action.

Ribavirin may be a pivotal combination partner in enhancing the efficacy of IFN-α-based therapy. Here, we demonstrated the additive anti-tumor effect of ribavirin in combination with IFN-α. A study of an anti-viral therapy has concluded that ribavirin enhances specific interferon-sensitive gene expression by amplifying the IFN-α JAK/STAT pathway; it has shown that STAT1 and STAT3 phosphorylation is higher in hepatocytes co-treated with ribavirin and IFN-α than after treatment with IFN-α alone [17]. Another possible mechanism is that ribavirin stimulates ERK1/2 and subsequently promotes p53 activity [18]. In addition, ribavirin upregulates the expression of IFN-a receptor in hepatocytes and augments interferon-stimulated gene induction [19, 20]. These mechanisms are at least partly supported by the results of our study. However, further investigation should be performed to understand the mechanisms by which the combination of ribavirin and IFN-α exerts a potent anti-tumor effect.

It has been reported that ribavirin has anti-tumor properties in different types of malignant tumors, such as head and neck squamous cell carcinoma (HNSCC) cell line FaDu, in breast cancer and AML patients [15, 20, 21]. In our study, we demonstrated for the first time that both murine and human RCC cell lines can respond to the treatment with ribavirin alone or a combination of ribavirin and IFN-α. These results are consistent with previously reported data. As an oncogene, eukaryotic translation-initiation factor 4E (eIF4E) is widely overexpressed in various cancers [22]. eIF4E, which is an element of the mammalian target of rapamycin (mTOR) pathway, has been considered an important target for ribavirin anti-tumor activity [21, 23]. Thus, the direct effect of ribavirin on RCC cells might be associated with eIF4E. Ribavirin might also enhance the efficacy of rapamycin analogues everolimus and temsirolimus in the treatment of renal cell carcinoma.

Apart from a direct effect on RCC cells, our study also confirmed that ribavirin reduced IL-10 production and increased TGF-β secretion, thus exerting an immunomodulatory effect in RCC-cell microenvironment. Our previous studies have indicated that IL-10, a negatively immunomodulatory cytokine, had the ability to expand regulatory T cells (Tregs) and induce their conversion [24, 25]. However, a recent clinical study has demonstrated that high TGF-β1 mRNA levels in peripheral blood in metastatic RCC patients are independently associated with favorable progression-free survival and overall survival. Thus, unlike IL-10, TGF-β appears to have an immune-promoting function [26]. More importantly, ribavirin has immunostimulatory effect (by multiple mechanism) in hepatitis C, such as enhancing proliferation of T effector cells, increasing production of IFN-Ɣ in Th1 cells, and reversing Treg-mediated suppression of T effector cells [4]. It has been also suggested that ribavirin might reverse immunosuppression in malignant tumors, particularly in RCC. All these data, in conjunction with our results, suggest that ribavirin alone or in combination with other agents might be useful in immunotherapy for RCC.

## Conclusions

Our study provides the evidence that ribavirin in combination with IFN-α can significantly inhibit the proliferation and migration, induce apoptosis, arrest the cell cycle at the G2/M phase, and decrease IL-10 production in the RCC cell lines. Moreover, this treatment has no toxic effects in normal cell line. We still need to conduct further investigation of the precise mechanism by which ribavirin directly suppresses RCC cells. Anti-tumor effects of ribavirin should also be carefully examined in a model of RCC. These are some of the aspects of this complex system currently studied in our laboratory.

## Materials and methods

### Cell line and cell culture

The study was approved by the Ethics Committee of Harbin Medical University (13016). This study used human RCC cell lines 786–0 cells, which were donated by Dr. Shen of the Department of Urology at The first affiliated hospital of Jilin University. Mice renal carcinoma cell lines Renca and HEK-293 cells purchased from American Type Culture Collection. Culture medium for Renca consisted of complete growth medium RPMI-1640 (Hyclone, Irvine, CA, USA) supplemented with heat-inactivated (56°C, 30 min) 10% fetal bovine serum (FBS, Gibco), 100 U/ml penicillin and 100 μg/ml streptomycin (Gibco). 786–0 cells were grown in Dulbecco’s modified Eagle’s medium (DMEM, Hyclone) containing 10% FBS (Hyclone) and 100 U/ml penicillin and 100 μg/ml streptomycin (Gibco). Above cells were cultured in an atmosphere of 5% CO2 in air at 37°C.

Ribavirin and interferon α. Ribavirin and recombinant mouse and human Interferon α (IFN-α) were purchased Tokyo Chemical Industry (TCI) and eBioscience, respectively.

### Effect of ribavirin alone, IFN-α alone or combination of ribavirin and interferon α on proliferation of renca and 786–0 cell lines

All cells were plated on 96-wells plates (5 × 10^3^ cells/well) incubated in respectively serum overnight at 37°C in a 5% CO_2_ incubator. After removal of media, 100 μl/well media containing different concentration of agents (ribavirin alone, IFN-α alone and combination of ribavirin and IFN-α) were added to each well. Plates were incubated for 72 h without subsequent media changed. Thereafter cells proliferation was measured using Cell Counting kit (CCK-8, Dojindo, Japan). Absorbance was measured at 450 nm. The OD values were measured to represent proliferation of cells. All the experiments were performed in triplicate.

### Assessment apotosis by annexin-V and propidium iodide

To evaluate effect of ribavirin alone, IFN-α alone or combination ribavirin and interferon α on renal cell carcinoma cells, annexin-V and propidium iodide (PI) (Apoptosis Detection Kit, annexin-V-FITC, BD, Biosciences Pharmingen, San Diego, CA) were used double stain technique, which was also used to distinguish between apoptosis (annexin-V positive, PI negative) and necrotic (annexin-V positive, PI positive) cells. 2 × 10^5^ cells under different culture condition were resuspended in 100 μl binding buffer, incubated with 2 μl Annexin-V-FITC (20 μg/ml) for 15 minutes on the ice in the dark, thereafter, each sample was incubated with 1 μl PI (50 μg/ml) for 2 minutes, and were then immediately analyzed by an Accuri C6 flow cytometer (BD Accuri Cytometers, MI). A minimum of 10,000 events was acquired for each sample.

### Cell cycle analysis by flow cytometry

To examine the effect of ribavirin and IFN-α on the renal cell carcinoma cells, we performed cell cycle analysis using above flow cytometry following manufacturer’s instruction. Briefly, the cells treated with ribavirin and IFN-α were stained with propidium iodide (PI) and were processed for cell cycle analysis.

### Cytotoxicity analysis of ribavirin and IFN-α with MTT assay

HEK-293 cells from American Type Culture Collection were seeded in 96-well flat bottom tissue culture plates at a density approximately 0.5-1 × 10^4^/well and were cultured. When the cells paved each bottom of well, the cells was incubated with or without ribavirin and IFN-α at different concentrations. After the cells were grown for 72 h, a volume 10 μl of the MTT reagent (Sigma, Hamburg, Germany) was added to each well. The plate was incubated for another 4 h at 37°C. After the MTT crystals were then solubilized with DMSO, absorbance of the cells was measured at 490 nm. Cytotoxity was expressed with OD value. All MTT experiments were performed in triplicates.

### Transwell migration assay

The assay was performed using the 24 well conning chamber plate with 8-μm pore size polycarbornate memberane filters. 1 × 10^5^/well cells were placed in the upper chamber containing DMEM with 10% FBS, the lower chamber also contained DMEM with 10% FBS. The plates with cells were incubated at 37°C in a 5% CO_2_ incubator for 48 h. After incubation, the cells on the memberane filter were fixed with 4% (v/v) formaldehyde for 10 minutes at room temperature. The samples were washed with phosphate buffered solution (PBS) and were stained with 0.5% crystal violet for 30 minutes. The samples were then washed (×3) with PBS. The cells on the upper surface of the filter removed with a cotton swab. The migratory cells were evaluated by counting the cells that migrated to the lower side of the filter by bright field microscopy at 200× magnification. Five random fields counted for each filter, and each sample was assayed in triplicate.

### Detection of IL-10 and TGF-β concentration in the supernatant by ELISA

To investigate effect IFN-α alone, ribavirin alone, and combination of IFN-α and ribavirin on production of IL-10 and TGF-β in the cell lines, we performed ELISA assay. The supernatant of each culture condition was collected. Thereafter all samples were measured using ELISA kit for IL-10 and TGF-β as per manufacturer’s instructions.

### Statistical analysis

All experiments were repeated multiple and the results were expressed as the mean ± SD. Statistical analysis was performed using SSPS 13.0 software. One-way ANOVA with a multiple comparison was used for data analysis. P < 0.05 was considered to be significant. To determine synergistic, additive or antagonistic effect of the combination of ribavirin and IFN-α, we take the method of interaction analysis using CompuSyn (ComboSyn, Inc). We used to calculate the combination index (CI). Synergy, additivity and antagonism are defined as CI < 1, CI = 1, CI > 1, respectively.
